# Effectiveness of self-re-learning using video recordings of advanced life support on nursing students’ knowledge, self-efficacy, and skills performance

**DOI:** 10.1186/s12912-021-00573-8

**Published:** 2021-03-31

**Authors:** Kyeongmin Jang, Sung Hwan Kim, Ja Young Oh, Ji Yeon Mun

**Affiliations:** 1 Department of Nursing, SMG-SNU Boramae Medical Center, 20, Boramae-ro 5-gil, Dongjak-gu, Seoul, Republic of Korea; 2 Department of Nursing, Chung-Ang University, 84 Heukseok-ro, Dongjak-gu, Seoul, Republic of Korea; 3 Department of Nursing Science, Ajou University, 164 Worldcup-ro, Yeongtong-gu, Wuwon, Republic of Korea

**Keywords:** Advanced life support, Retention of skills performance, Nursing students

## Abstract

**Background:**

Nurses are presumably the first to see an in-hospital cardiac arrest patient. This study proposed measuring nursing students’ knowledge, self-efficacy, and skills performance in advanced life support (ALS), 6 months after training, by sending videos taken during their final skills test after the ALS training.

**Methods:**

This is an experimental study using a randomised control group design. This study was conducted from June to December 2018, and the subjects of the study were 4th year students, recruited through a bulletin board at a nursing university. The participants’ knowledge, self-efficacy, and skill performance in ALS were evaluated immediately after the training, and participants were videotaped during the final skills test. Thereafter, the videos were sent to the experimental group through a mobile phone messenger application, once a month, from the third month after training. Approximately six months after training day, a follow-up test was conducted for the measured variables using a blinded method. The paired t-test and Wilcoxon signed-rank test were used to compare the two groups pre-and post-intervention. The statistical significance level was set at *p* < .05.

**Results:**

Six months after the ALS training, knowledge scores decreased significantly in both groups (*p* < 0.001). Self-efficacy decreased by about 3 points from 50.55 to 47.18 in the experimental group (*p* = 0.089), while it decreased by 10 points in the control group, from 50.67 to 39 (*p* < 0.001). The skills performance decreased from 27.5 to 26.68 in the experimental group, while it decreased significantly from 27.95 to 16.9 in the control group (*p* < 0.001).

**Conclusion:**

Self-study with videos taken during an ALS skills test helps enhance the sustainable effects of training such as knowledge, self-efficacy, and skills performance.

## Background

Cardiac arrest accounts for 80% of deaths in hospitals, and survival rates are not significantly different from those of pre-hospital cardiac arrests [1]. In a study conducted in a tertiary medical institution in Korea [2], the survival rate of cardiac arrest patients in the hospital for 24-h survival and survival discharge was 23.7 and 6.4%, respectively. These findings may be related to the healthcare provider’s ability to perform CPR, in addition to the patient’s age and health status.

Nurses are more likely to detect a cardiac arrest early in their care by observing the patient’s condition and its changes over 24 h [3]. Therefore, nurses’ early response is crucial [4]. When a cardiac arrest occurs in a hospital, Advanced Life Support (ALS), including Basic Life Support (BLS), monitor use, emergency medication, and advanced airway maintenance, is performed [5]. During treatment, the necessity of defibrillation and the drugs to be administered vary depending on the electrocardiogram (ECG) rhythm [6, 7]. Therefore, nurses require ALS training. Specifically, nursing students who will become future nurses are more likely to encounter cardiac arrest patients for the first time; therefore, training them in ALS is essential [8]. In addition, nursing students should be ready to work without fear when encountering cardiac arrest patients while working in the hospital after obtaining qualifications.

Previous studies on the effects of ALS training revealed that simulation-based ALS training can improve nursing students’ knowledge, critical thinking, and problem-solving [9, 10] and can also enhance their self-confidence and clinical performance [11]. Therefore, it is recommended that nursing students conduct ALS training in the nursing department curriculum. By doing so, when nursing students become new nurses and encounter cardiac arrest patients in actual clinical practice, it is important to continue to maintain knowledge and skills so that they can successfully perform their role as members of the resuscitation team [12]. However, three to six months after ALS training, the effect decreases [13, 14], and retraining is required to maintain its efficacy [15, 16]. Although prior studies have shown the effectiveness and persistence of ALS, most of these are cross-sectional studies that confirm the effects before and after education. Intervening research is required to confirm the continuity of educational effects and to maintain knowledge, self-efficacy, and performance.

Therefore, this study compared the continuous effects of ALS training by measuring nursing students’ knowledge, self-efficacy, and skills performance in ALS, immediately after training and six months after training.

## Methods

### Design and setting

This experimental study, using a randomised control-group pre-test–post-test design, verified the sustained effects of the Korean Advanced Life Support (KALS) training on nursing students’ knowledge, self-efficacy, and skill performance in ALS.

### KALS provider course

KALS training is an ALS training program developed by the Korean Association of Cardiopulmonary Resuscitation (KACPR) ALS committee, since factors such as long training hours and the high training costs of the American Heart Association’s (AHA ACLS Provider Course obstruct the spread of education. This training is a one-day (five to six-hour) course wherein the knowledge and skills required for first aid treatment of cardiac arrest patients in hospitals or ambulances is provided.

### Participants

In May 2018, an announcement was made on the bulletin board of the department of nursing at a university about the annual ALS training and recruited the study participants. The study participants were 4th-year students in the Department of Nursing at K University, who met the following inclusion criteria: 1) understood the research purpose, participated voluntarily, and agreed to shoot videos, 2) had completed the AHA’s BLS training as a final year student, and 3) who used a mobile phone messenger application that could send videos. Participants who refused to shoot videos or had already completed the AHA’s Advanced Cardiovascular Life Support (ACLS) provider course were excluded from the study because of differences in the parameters to be measured.

### Sample size

The number of study participants was calculated using the G-Power (ver. 3.1.9) program. For the t-test, a statistical method was used to compare the means of two groups; the minimum number of participants was 21 per group. Therefore, based on similar prior studies [17, 18], considering a dropout rate of 20%, 50 participants were recruited, and 25 participants were assigned to each group. Allocation concealment was applied to the experimental and control groups, and the participants were not informed about which group they belonged to until the post-test. After the post-test, the control group was also sent a video of the final skills test through the mobile messenger application.

### Procedure

**Fig. 1:**
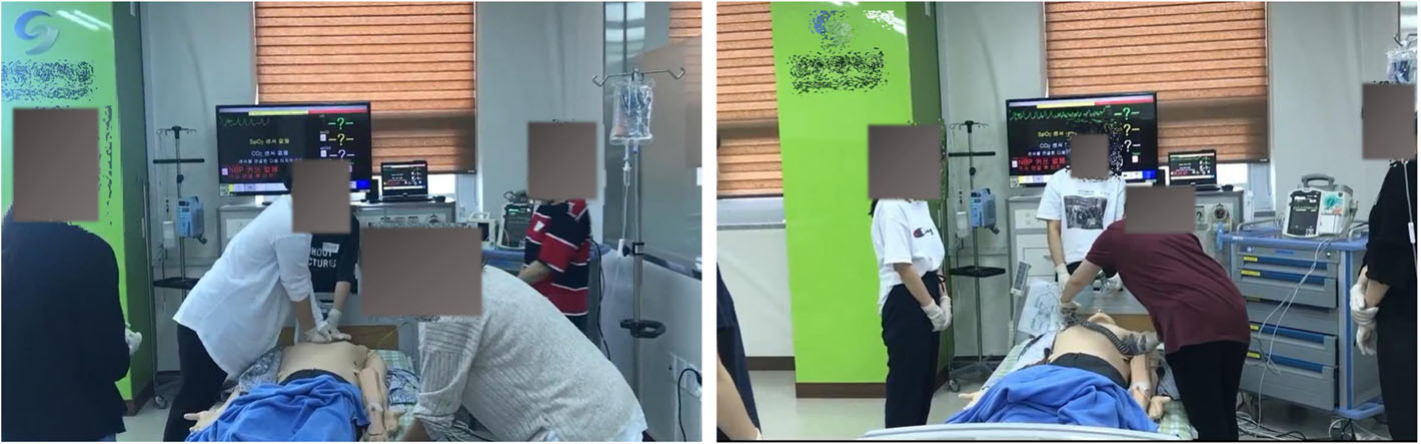
Screenshots of recorded videos during skill performance evaluation

The study followed the CONSORT 2010 guidelines [19]. The ALS training was conducted in the simulation lab at the nursing university to which the research participants belonged and was conducted over five days: 3, 9, 10, 16 and 17 June 2018. Training hours were from 09:00 to 15:00, including one hour for lunch. The study participants’ ALS training was conducted by four professional instructors registered in KACPR and consisted of five to six students per group. Two instructors were assigned to each team. One instructor conducted the final skills evaluation, and the other recorded a video using a mobile phone (iPhone 8, Apple Inc.) during the skill evaluation (Fig. 1). Immediately after ALS training, all participants were surveyed for ALS knowledge and self-efficacy and evaluated for ALS skill performance. For randomisation, after ALS training was conducted on all study subjects, a research assistant who did not participate in the experimental intervention was randomly assigned 25 subjects each to the experimental group and the control group, using a random allocation program at https://www.randomizer.org. The allocation was concealed from the experimental group and the control group until the post-test.

Each participant’s recorded video of their skills test process was sent to the experimental group through a mobile messenger application (KakaoTalk messenger, Kakao Corporation) once a month from the third month after training, while no arbitration was conducted on the control group. To prevent information leakage between the intervention and control groups, study participants were urged to not discuss whether they received videos. About six months after the date of the initial training, two evaluators participated in the evaluation in the same way and conducted a post-test of the measurement variables without knowing the experimental and control groups.

**Fig. 2:**
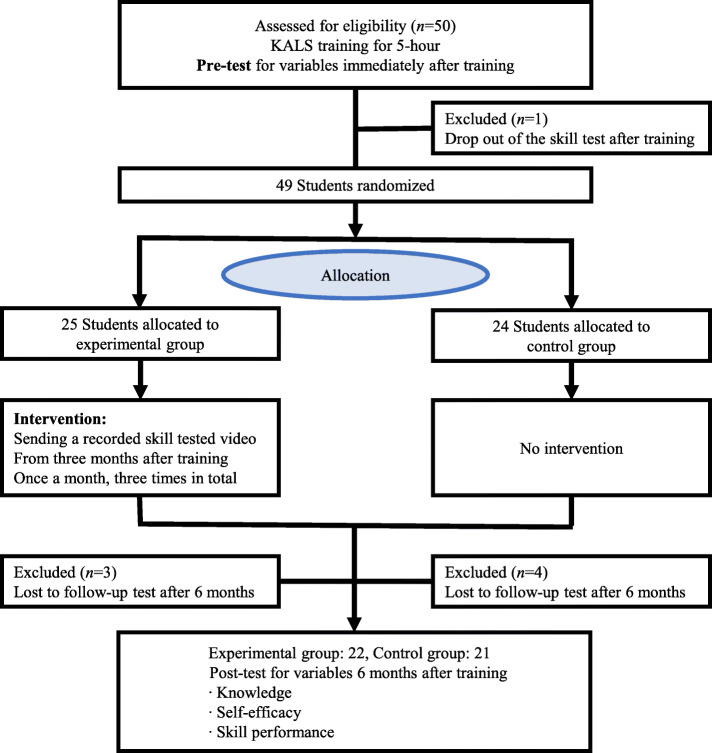
Study process

The flow chart of the research process is shown in Fig. 2.

### Primary outcomes

#### ALS knowledge

The ALS knowledge measuring tool was developed by the researcher based on the content of the ACLS provider manual and the 3rd edition of the KALS textbook by the KACPR KALS committee. The validity of the knowledge was evaluated by one emergency physician, two ACLS and KALS instructors, two nurses with more than ten years of emergency room experience, and two nursing professors. All items had a Content Validity Index (CVI) of 0.8 or higher. It comprised four questions on BLS, five on ECG recognition, four on teamwork, four on ACLS, and three on post cardiac arrest care (PCAC). A total of 20 points are possible, and the higher the score, the higher the knowledge score.

#### ALS self-efficacy

In this study, resuscitation self-efficacy [20] was measured by ALS self-efficacy using a modified and supplemented tool. The revised tool comprised 12 questions, including 2 questions on BLS, 3 on ECG recognition, 2 on teamwork, 3 on ALS, and 2 on PCAC. Each item was rated on a five-point Likert scale with 5 points for ‘very confident’ and 1 point for ‘very unconfident’. The higher the score, the higher the self-efficacy for professional resuscitation. The tool’s internal reliability at the time of development had a Cronbach’s alpha value of 0.91 [20] and 0.87 in this study. A total of 60 points are possible, with the lowest being 12. The higher the score, the higher the ALS self-efficacy.

#### ALS skills performance

The Training of In-hospital Cardiac Arrest (TROICA) checklist of the KALS committee, developed for the KALS provider course, was used after obtaining the KACPR ALS committee’s consent to measure ALS skills. The TROICA, a measurement tool for KALS skills, comprises 15 questions, including 2 questions on BLS skills, 3 on teamwork, 3 on ALS algorithms, 5 on cardiac arrest cognition and appropriate treatment instructions, and 2 on post-cardiac care. Each question was scored two, one, and zero points for correct, insufficient, and incorrect performance, respectively. A total of 30 points are possible, and the higher the score, the higher the ALS skill performance.

### Ethical consideration

This study was approved by the Institutional Review Board committee of the hospital to which the first author belongs (IRB approval number: 20180518 / 20–2017-33 / 062). The purpose and procedures of the study were explained to the participants, and informed consent was obtained in writing from the voluntary participants. In further consideration of ethics, the recorded video of their ALS skills test process was also sent to the control group after the study.

### Statistical analysis

Collected data were analysed using SPSS 24.0 (for Windows), and the selected statistical significance level for hypothesis testing was *p* < 0.05. The general characteristics of the experimental and control groups were analysed using descriptive statistics of frequency, percentage, mean, and standard deviation. To test the normality of the measured variables, the participants were analysed using the Shapiro-Wilk test, which is mostly used for 3 to 50 participants [21]. The t-test and Mann-Whitney U test were used to verify the general characteristics of the experimental and control groups, and the homogeneity of the dependent variables before the experiment. To confirm the pre-post change of the experimental group and the control group, the normal distribution was analysed by paired t-test, and the non-normal distribution was analysed using the Wilcoxon signed-rank test. The reliability of the measurement tool was analysed using Cronbach’s alpha.

## Results

### General characteristics of participants and homogeneity test of the experimental and control groups

Three of the 25 participants in the experimental group failed to follow up on a personal schedule, and 4 of the 25 participants in the control group dropped out of contact. Therefore, the analysis included 22 and 21 participants in the experimental and control groups, respectively (Fig. 2). There was no significant difference between the experimental and control groups’ homogeneity test for general characteristics, including gender, age, average grades, nursing degree satisfaction, and university life satisfaction (Table 1).

**Table 1:** Homogeneity Test of General Characteristics

Characteristics		Exp. (*n* = 22)	Cont. (*n* = 21)	*X*^2^ or F	*p*
		*n* (%) or mean ± SD	*n* (%) or mean ± SD		
Sex	Male	5(22.7)	2(9.5)	1.374	.412
	Female	17(77.3)	19(90.5)		
Age (years)	Under 22	13(59.1)	16(76.2)	.304	.694
	22 years or older	9(40.9)	5(23.8)		
	mean ± SD	22.82 ± 4.25	22.3 ± 5.33		
Average Credits	Under 3.0	1(4.5)	1(4.8)	.75	.687
	3.0 ~ 3.9	18(81.8)	15(71.4)		
	4.0 or higher	3(13.6)	5(24.8)		
Satisfaction of Nursing	Under 60	4(18.2)	1(4.8)	.027	.353
	60 ~ less than 80	10(45.5)	11(52.4)		
	80 or higher	8(36.3)	9(42.8)		
	mean ± SD	67.73 ± 11.10	70.95 ± 10.44		
Satisfaction of College life	Under 60	4(18.2)	1(4.8)	.095	.14
	60 ~ less than 80	13(59.1)	14(66.6)		
	80 or higher	5(22.7)	6(28.6)		
	mean ± SD	73.18 ± 12.87	75.71 ± 12.07		

*Cont.* control group, *Exp.* experimental group, *SD* standard deviation

### Normality and homogeneity test of experimental and control groups

In the Shapiro-Wilk test of normality, the sub-items of ALS knowledge and ALS skills performance did not show a normal distribution; however, the sub-items of ALS self-efficacy showed a normal distribution. The homogeneity test using the Mann-Whitney U test and t-test showed that the average score in ALS knowledge (*p* = .041) for the experimental group (3.09) was lower than that of the control group (3.48). Additionally, there were no significant differences in knowledge scores, self-efficacy, and skills performance, ensuring homogeneity between the groups (Table 2).

**Table 2:** Homogeneity Test of Dependent Variables

Variable		Exp. (*n* = 22)		Cont. (*n* = 21)		t/Z	*p*
		Mean	SD	Mean	SD		
Knowledge	BLS	3.77	.429	3.86	.359	−.703	.482
	ECG Recognition	3.95	.653	4.1	.7	−.917	.359
	Teamwork	3	.535	3	.707	0	1
	ACLS	3.09	.61	3.48	.602	−2.044	.041
	PCAC	2.73	.456	2.71	.463	−.094	.925
	Total	16.68	1.129	17.19	1.078	−1.545	.122
Self-Efficacy	BLS	12.64	1.364	12.29	1.231	−.751	.453
	ECG Recognition	8.32	1.323	8.48	.981	−.191	.848
	Teamwork	12.5	1.793	12.86	1.459	−.811	.417
	ACLS	8.41	1.26	8.38	1.071	−.038	.97
	PCAC	8.64	1.217	8.86	.964	−.494	.621
	Total	50.55	6.085	50.67	4.83	−.072	.943
Skill Performance	BLS	4	.001	4	.001	0	1
	ECG recognition	5	.756	5.33	.913	−1.727	.084
	Teamwork	5.64	.727	5.76	.625	−.681	.496
	ACLS	9.27	.767	9.14	.854	−.445	.656
	PCAC	3.5	.598	3.71	.463	−1.211	.226
	Total	27.5	.74	27.95	1.071	−1.306	.192

*ACLS* advanced cardiovascular support, *BLS* basic life support, *Cont.* control group, *ECG* electrocardiogram, *Exp.* experimental group, *PCAC* post cardiac arrest care, *SD* standard deviation

### Comparison of dependent variables immediately and six months after training in the experimental and control groups

Table 3 shows the differences between the experimental and control groups’ knowledge, self-efficacy, and skills performance immediately after and six months after training. Regarding the total knowledge score, the experimental group’s pre-test score (16.68) showed a statistically significant decrease compared to the post-test score (15.32) (*p* = .001), and the control group’s pre-test score (17.19) also showed a significant decrease compared to the post-test score (14.67) (*p* < .000). Self-efficacy and skills performance scores were reversed. Regarding the total self-efficacy score, the test group’s pre-test score (50.55) was not significantly lower compared with the post-test score (47.18), but the control group’s pre-test score (50.67) was significantly lower compared with the post-test score (39). Regarding the skills performance score, the pre-test score of the experimental group (27.5) was not significantly lower decreased compared to the post-test score (26.68), but the pre-test score of the control group (27.95) was significantly lower than the post-test score (16.9).

**Table 3:** Comparison of Variables between Experimental and Control Groups after 6 Months of Training

Variables		Exp. (*n* = 22)		Cont. (*n* = 21)		t/Z	*p*
		Mean	SD	Mean	SD		
Knowledge	BLS	3.32	.646	3.05	.498	−1.606	.108
	ECG recognition	4.00	.756	3.76	.944	−.773	.439
	Teamwork	2.45	.739	2.57	.978	−.291	.771
	ACLS	3.14	.640	2.62	.921	−1.905	.057
	PCAC	2.50	.598	2.67	.577	−1.061	.289
	Total	15.32	1.460	14.67	1.278	−1.401	.161
Self-efficacy	BLS	12.36	1.814	10.48	2.581	−3.125	.002
	ECG recognition	7.55	1.711	6.38	2.037	−1.882	.060
	Teamwork	11.73	2.028	9.05	3.138	3.341	.002
	ACLS	7.50	1.655	5.90	2.385	−2.325	.02
	PCAC	8.05	1.495	7.19	1.692	−2.025	.043
	Total	47.18	7.719	39.00	10.918	2.848	.007
Skill performance	BLS	3.86	.468	2.52	.981	−4.776	.001*
	ECG recognition	5.59	.503	3.62	1.024	−5.250	.001*
	Teamwork	5.23	1.066	1.67	.658	−5.711	.001*
	ACLS	8.68	1.729	6.81	1.537	−3.481	.001*
	PCAC	3.32	.716	2.24	.768	−4.123	.001*
	Total	26.68	3.107	16.90	3.520	−5.267	.001*

*ACLS* advanced cardiovascular support, *BLS* basic life support, *Cont.* control group, *ECG* electrocardiogram, *Exp.* experimental group, *PCAC* post cardiac arrest care, *SD* standard deviation; * = *p* < .001

### Comparison of dependent variables six months after training between experimental and control groups

**Fig. 3:**
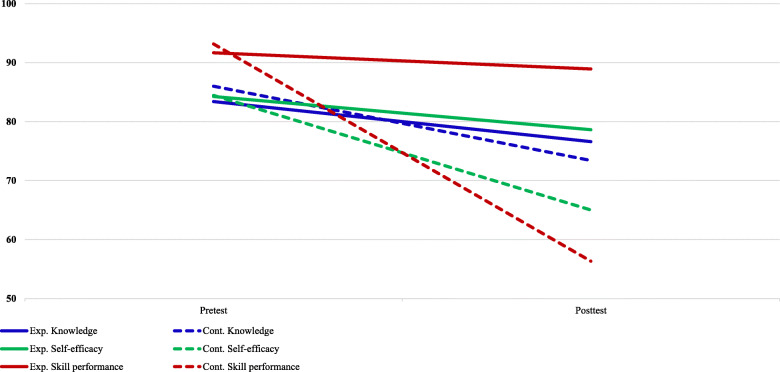
Differences of variables between pre-test and post-test in both groups

The knowledge, self-efficacy, and skills performance of the experimental and control groups were examined six months after training. The experimental group scored higher on the total score than the control group; however, there was no significant difference in the sub-items. The self-efficacy score of the experimental group was higher than that of the control group for all sub-items. The ECG reading of the sub-items was higher in the experimental group (7.55) than in the control group (6.38), but there was no significant difference (*p* = .06). The other sub-items included BLS: 12.36 vs. 10.48, Teamwork: 11.73 vs. 9.05, ACLS: 7.50 vs. 5.90, PCAC: 8.05 vs. 7.19, and Total: 47.18 vs. 39. At 39.00 points, the experimental group was significantly higher than the control group (*p* = .002, p = .002, *p* = .02, *p* = .043, and *p* = .007, respectively). In the skills performance score, the experimental group scored higher than the control group in all sub-items (*p* < .001) (Table 4; Fig. 3).

**Table 4:** Differences between Pre-test and Post-test in Experimental Group and Control Group

Variable			Pre-test		Post-test		t/Z	*p*
			Mean	SD	Mean	SD		
Knowledge	BLS	Exp.	3.77	.429	3.32	.646	−2.352	.019
		Cont.	3.86	.359	3.05	.498	−3.69	.001*
	ECG recognition	Exp.	3.95	.653	4	.756	−.233	.816
		Cont.	4.1	.7	3.76	.944	−1.393	.163
	Teamwork	Exp.	3	.535	2.45	.739	−2.546	.011
		Cont.	3	.707	2.57	.978	−1.651	.099
	ACLS	Exp.	3.09	.61	3.14	.64	−.258	.796
		Cont.	3.48	.602	2.62	.921	−2.797	.005
	PCAC	Exp.	2.73	.456	2.5	.598	−1.387	.166
		Cont.	2.71	.463	2.67	.577	−.302	.763
	Total	Exp.	16.68	1.129	15.32	1.46	−3.256	.001
		Cont.	17.19	1.078	14.67	1.278	−3.715	.001*
Self-efficacy	BLS	Exp.	12.64	1.364	12.36	1.814	−.461	.645
		Cont.	12.29	1.231	10.48	2.581	−2.702	.007
	ECG recognition	Exp.	8.32	1.323	7.55	1.711	1.771	.091
		Cont.	8.48	.981	6.38	2.037	−3.438	.001
	Teamwork	Exp.	12.5	1.793	11.73	2.028	−1.549	.121
		Cont.	12.86	1.459	9.05	3.138	−3.839	.001*
	ACLS	Exp.	8.41	1.26	7.5	1.655	−1.907	.057
		Cont.	8.38	1.071	5.9	2.385	−3.297	.001
	PCAC	Exp.	8.64	1.217	8.05	1.495	−1.34	0.18
		Cont.	8.86	.964	7.19	1.692	−3.349	.001
	Total	Exp.	50.55	6.085	47.18	7.719	1.781	.089
		Cont.	50.67	4.83	39	10.918	4.489	.001*
Skill performance	BLS	Exp.	4	.000a	3.86	.468	−1.342	.18
		Cont.	4	.000a	2.52	.981	−3.8	.001*
	ECG recognition	Exp.	5	.756	5.59	.503	−2.372	.018
		Cont.	5.33	.913	3.62	1.024	−3.69	.001*
	Teamwork	Exp.	5.64	.727	5.23	1.066	−2.07	.038
		Cont.	5.76	.625	1.67	.658	−4.084	.001*
	ACLS	Exp.	9.27	.767	8.68	1.729	−1.312	.19
		Cont.	9.14	.854	6.81	1.537	−3.662	.001*
	PCAC	Exp.	3.5	.598	3.32	.716	−.775	.439
		Cont.	3.71	.463	2.24	.768	−3.919	.001*
	Total	Exp.	27.5	.74	26.68	3.107	−1.021	.307
		Cont.	27.95	1.071	16.9	3.52	−4.021	.001*

*ACLS* advanced cardiovascular support, *BLS* basic life support, *Cont.* control group, *ECG* electrocardiogram, *Exp.* experimental group, *PCAC* post cardiac arrest care, *SD* standard deviation; * = *p* < .001

## Discussion

This study verified the effectiveness and retention of nursing students after ALS training. Consequently, the experimental group that received the video showed higher persistence in knowledge, self-efficacy, and skills performance than the control group. Based on these results, ways to increase and maintain the effectiveness of ALS training are discussed.

In a study [22] evaluating BLS and ACLS knowledge of healthcare providers, participants’ average score did not exceed 50%. However, healthcare providers with BLS or ACLS training had higher knowledge scores than those without. Therefore, ALS training can improve knowledge scores. For nursing and medical school students, a study [14] measuring knowledge scores immediately after, 3–6 months after, and 6–9 months after, using traditional ACLS training and high-fidelity mannequins, showed that knowledge scores measured after 3–6 months and 6–9 months were lower compared to knowledge scores immediately after training. Additionally, a study [23] comparing existing and high-fidelity simulations on the persistence effect of ACLS knowledge among medical students also reported that ACLS knowledge scores decreased significantly after one year compared to immediately after training. In the results of this study, the knowledge scores measured after six months decreased in both the control and experimental groups, but the experimental group showed a smaller decrease than the control group. These results are similar to those of a previous study [10] on nursing students in which the control and experimental groups underwent traditional, lecture-based ACLS training and simulation-based ACLS training, respectively. Simulation training was more effective in acquiring and maintaining ACLS knowledge than lecture-based education. In a study [24] measuring knowledge two and eight weeks after ACLS training for nurses in the emergency department, knowledge retention eventually decreased. The authors emphasise that knowledge retention could be increased through simulation-based re-learning after ACLS training. Reduction of knowledge after ACLS training is considered a natural result over time, and an education renewal program is necessary to maintain knowledge retention. Additionally, it may be possible to consider ways to maintain the knowledge level through newsletters and email notifications.

Self-efficacy is an individual’s belief in his/her ability to perform a specific task or activity [25]. Simulation-based ACLS training can increase self-efficacy in ACLS skills performance. In a study [26] measuring self-efficacy before and after simulation-based ACLS training, undertaken by medical students, self-efficacy increased significantly after training. Furthermore, the experimental [10] group showed significantly higher self-efficacy than the control group in a study comparing the experimental group with the simulation-based ACLS training, and the control group with the traditional lecture-based resuscitation training. However, self-efficacy persistence decreased significantly over time [14]. In a study [27] comparing healthcare efficacy immediately after and six months after Paediatric Advanced Life Support (PALS) training of healthcare providers, self-efficacy measured after six months was significantly lower compared to immediately after training. Additionally, in a study [28] comparing nursing students immediately after and three months after BLS training, self-efficacy decreased significantly after three months.

The self-efficacy of nursing students can be determined by the ongoing interaction between cognitive, behavioural, and environmental factors [29]. The self-efficacy measured immediately after the training in this study showed no difference between the control and experimental groups. Although the self-efficacy measured six months after the training was not significantly lower in the experimental group, it was significantly lower in the control group. These results suggest that they could have re-learned their knowledge and skills of ACLS by watching their skills test videos, which could improve their retention of self-efficacy.

Simulation-based ALS training is not highly effective in improving skills performance based on previous studies. However, this performance ability decreases rapidly over time [30]. Only 30% of the nurses passed the skills test measured three months after ALS training [31], and other studies [32, 33] reported a decrease in ALS skills performance six months after ALS training. Furthermore, a systematic literature review of skills performance retention following ALS training by healthcare providers showed a decrease in skills performance between six months and one year after training [34]. Comparing the skills performance of the control group, which had no intervention in this study, immediately after and six months after training, the skills performance measured six months after training decreased significantly. As such, skills performance begins to decline between three and six months and appears to decrease significantly after one year. As a method of retaining skills performance, iterative simulation-based ALS training can improve retention [34, 35]. After six months of clinical experience, training results showed a longer-lasting effect on skills performance than those without clinical experience [34, 36]. Moreover, in ACLS training, practicing for a 2-min cycle, similar to the actual time, resulted in higher skills performance measured three months after compared to short training [37].

In this study, the experimental group was sent a video of their final skills test three months later, a relatively simple and cost-effective method to retrain themselves. Six months later, the experimental group that received the video had better retention of skills performance than the control group. This video delivery method can increase the retention of skills performance and retraining time. Timely reminders to participants who have received ALS training will be required for self-retraining.

### Limitations

This study was a randomised control study; however, there are some limitations. First, this study’s results are difficult to generalise because the sample size was small, and the experiment was conducted in one institution. Second, since the same standardised tool was used at each data collection point, participants could have possibly remembered previous answers. Lastly, this local cross-sectional study of nursing students cannot be generalised to Korea. Further trials are required to improve the retention of ACLS knowledge, self-efficacy, and skills performance.

## Conclusion

This study showed that sending videos to nursing students during their final skills test between training and three to six months afterwards—a relatively simple and cost-effective method after ALS training to induce self-learning, can be effective in retaining knowledge, self-efficacy, and skills performance for ALS—Further studies should confirm the most effective timing for sending the videos. Replication studies are required to further confirm these findings.

## Data Availability

The datasets used and/or analysed during the current study are available from the corresponding author upon reasonable request.

## Abbreviations

ALS: Advanced life support
CPR: Cardiopulmonary resuscitation
BLS: Basic life support
ECG: Electrocardiogram
KALS: Korean advanced life support
ACLS: Advanced cardiovascular life support
KACPR: Korean Association of Cardiopulmonary Resuscitation
AHA: American Heart Association
CVI: Content Validity Index
PCAC: Post cardiac arrest care
TROICA: Training of In-hospital Cardiac Arrest
